# Prevalence and predictors of insomnia and its treatment-seeking among older adults in India

**DOI:** 10.1186/s44167-024-00044-w

**Published:** 2024-02-01

**Authors:** Manas Ranjan Pradhan, Daisy Saikia

**Affiliations:** 1 Department of Fertility and Social Demography, International Institute for Population Sciences (IIPS), Mumbai, India; 2 Research Scholar, International Institute for Population Sciences (IIPS), Govandi Station Road, Deonar, Mumbai, Maharashtra 400088 India

**Keywords:** Insomnia, Older adults, Predictors, Treatment-seeking, India

## Abstract

**Background:**

Insomnia is a serious health problem among older adults and, if untreated, is linked to a high morbidity rate and decreased quality of life. There is limited empirical evidence on Insomnia and its treatment-seeking exclusively among older adults (60 plus years) using representative data in India. This study assesses the prevalence and predictors of Insomnia and its treatment-seeking among older adults.

**Methods:**

Data gathered through the nationally-representative Longitudinal Ageing Study in India (LASI); Wave 1 (2017-18) was used for the analysis. Specifically, information from older adults aged 60 and above for whom complete information on insomnia was available (n- 31,464) was considered for the analysis. Binary logistic regression was used to check the adjusted effects of insomnia’s socio-demographic and economic predictors and its treatment-seeking status. Stata was used for the data analysis with a 5% significance level.

**Results:**

37% of older adults had insomnia. Increasing age, female gender, living without a spouse, illiteracy, chronic health conditions, nutritionally underweight, physically inactive status, lack of exposure to mass media, Hindu religion, non-tribal status, and rural residence were significantly associated with insomnia. 3% of older adults sought treatment for insomnia. Not seeking treatment for insomnia was associated with male gender, exposure to mass media, physical activity, lack of chronic health issues, tribal status, living in a rural area, and being economically disadvantaged.

**Conclusions:**

A sizable number of older adults have insomnia, and the prevalence varies by their socioeconomic, demographic, and health status. Many modifiable risk factors like low education, chronic health conditions, smoking, being underweight, physical inactivity, and lack of exposure to mass media are identified. Treatment-seeking for Insomnia is further inadequate, enhancing the older adult’s vulnerability to various morbidities. Policy and program intervention to raise awareness about insomnia, including early identification and pharmacological and non-pharmacological treatment, will ensure better health and welfare of older adults. Estimations are based on self-report questionnaires; therefore, the possibility of recall bias and under-reporting cannot be ignored. Moreover, the estimation of insomnia may vary depending on various clinical definitions. However, a large sample size from a recent nationally representative survey with a robust sampling design is the strength of this study.

## Background

Sleep disorders affect a considerable population worldwide [1, 2]. One significant component of sleep disorders is insomnia [3, 4], which makes it difficult for people to fall and stay asleep. The International Classification of Sleep Disorders defines chronic insomnia as trouble falling asleep or staying asleep at least three times per week for three months despite having access to appropriate sleep opportunities [5]. The Diagnostic and Statistical Manual of Mental Disorders-5 subtypes insomnia as episodic if it lasts less than one month and as persistent if it lasts longer than three months [6]. Evidence worldwide indicates that 10–30% of people have chronic insomnia, with some estimates reaching 50–60% [7]. Short-term or chronic insomnia commonly affects older adults [8]. Among older adults, the overall prevalence of insomnia symptoms ranges from 30 to 48% [9].

Insomnia is a serious health problem among older adults due to its linkage with worsening general health, reduced quality of life, and morbidities, such as falls, institutionalization, and cognitive impairment [10]. Age-related biological changes that result in less deep sleep, more fragmented sleep, and early morning wakeups are one theory of why older adults are more likely to experience insomnia. Retirement-related lifestyle changes, poor physical function, polypharmacy, and an increasing burden of health issues are some of the precipitating factors, while social isolation, caregiving, and bereavement are some of the perpetuating factors [8]. Insomnia also confers an increased risk of suicidal tendencies [11]. Additionally, insomnia is associated with heart disease, hypertension, myocardial infarction and can further lead to metabolic syndrome and prostate cancer [9]. Insomnia doubles the chance of developing depression and hypertension [12]. Past studies found that women, the unemployed, widowed, separated, divorced, or single, those with a low education level, those with low socioeconomic status, and older adults are more prone to insomnia [13, 14]. Other variables associated with higher rates of insomnia include smoking [13], drinking [13], watching television [15], and a lack of physical activity [13]. A study revealed that religious involvement might improve sleep quality by reducing the mental, chemical, and physical arousal from psychological discomfort, substance use, stress exposure, and allostatic load, thus making it a social determinant of sleep [16]. Insomnia further increases with increasing age [17]. If left untreated, insomnia is linked to a high morbidity rate [9]. Recognizing and treating insomnia may improve the individual performance of older adults and prevent cardiovascular diseases, psychological difficulties, and other chronic conditions [4].

The importance of sleep for older adults’ overall health and well-being is well-researched and is becoming more widely recognized [18]. There are currently 60 million older adults in India, and this number is expected to rise to over 227 million by 2050 [19]. Thus, the burden of sleep-related health disorders is expected to increase as the older adult population grows. Available Indian studies on insomnia have important limitations, such as including only a narrow geographical region [3, 4], including only specific groups, such as outpatient department (OPD) patients [7], or including younger adults [17]. To the authors’ knowledge, no empirical study has employed a nationally representative sample and focused on insomnia and treatment-seeking among older adults (60+) in India. Against this backdrop, this study assesses the prevalence and predictors of insomnia and treatment-seeking among Indian older adults. Our results will inform programs and policy interventions aiming to improve the health of older adults in India and help achieve United Nation’s Sustainable Development Goal 3, which aims for health for all.

## Methods

### Data

The present study used data from Wave 1 of the nationally-representative Longitudinal Ageing Study in India (LASI) study conducted from 2017 to 18. A total of 72,250 older adults aged 45 + years and their spouses (irrespective of age) across India’s states and Union Territories (UTs) were surveyed. The LASI provides in-depth information on aging, economic aspects, social relationships, social support, family and life satisfaction, and health status, including sleeping disorders. The LASI used a multistage clustering sampling design to choose respondents for the survey. The first stage in each state involved selecting primary sampling units (PSUs), which were sub-districts; the second stage involved selecting secondary sampling units (SSUs), which were villages in rural and wards in urban areas of the selected PSUs. In the third stage, households were chosen from a list of villages in rural areas; in urban areas, sampling involved an additional stage—one Census Enumeration Block (CEB) was randomly selected in each urban area. In the fourth stage, households were selected from these CEBs. All men and women aged 45 and above and their spouses were interviewed in selected households.

The data were collected by qualified research investigators who conducted computer-assisted personal interviews (CAPIs). Interviews were only conducted with those individuals who gave informed consent and agreed to participate. The Indian Council of Medical Research (ICMR) provided guidance and ethical approval for conducting the LASI. The overall household response rate was 95.8%, and the individual response rate was 87.3% for all older adults aged 45 + years and their spouses. The published survey report provides a detailed sampling design, data collection tools and processes, and quality control measures [20]. In the present study, we examined data from older adults aged 60 + for whom complete information on insomnia was available (*n* = 31,464).

### Outcome variables

The outcome variables were (i) having insomnia (no, yes) and (ii) seeking treatment for insomnia (no, yes). The LASI gathered information on sleep quality and whether medications or other forms of treatment were used for one month before the survey date. Specifically, it captured the frequency of trouble falling asleep, waking up during the night and having difficulty falling back to sleep, and waking too early in the morning and not being able to fall asleep again. Additionally, the survey asked whether the respondent had taken any medications or treatment to promote sleep in the past month. Several variables have been used for estimating insomnia in different studies and contexts. In the present analysis, older adults aged 60 and above years were considered to have insomnia if they had self-reported any of the following: (a) trouble falling asleep three or more nights per week, or (b) waking up at night three or more nights per week, or (c) waking up too early in the morning three or more nights per week, or (d) took any medications or treatment to promote sleep in the past month. The definition of insomnia used in this study conforms with standard international literature defining insomnia [5, 6], given the available data in the LASI Survey. The survey asked a question, i.e., in the past month, did the respondents take any medications or use other treatments to help them sleep? Respondents answering “Yes” to this question were considered to have been seeking treatment for insomnia in this study.

### Predictor variables

The individual, household, and community-level predictors used in the analysis were chosen based on literature review [3, 4, 10] and information available in the LASI. Participant characteristics included in the analysis were age in years (60–74,youngest-old, 75–116, middle/oldest-old), gender (male, female), living arrangement (living alone or with only others—distant relatives or non-relatives, living with spouse and children/ distant relatives or non-relatives, and living with children and distant relatives or non-relatives), education (illiterate, literate), smoking status (yes, no), mass media exposure (yes, no), physical activity (no/light, moderate, vigorous), body mass index (BMI; underweight ≤ 18.4 kg/m2; normal 18.5 to 24.9 kg/m2; overweight/obese ≥ 25 kg/m2) [21], cardiovascular diseases (CVD; yes, no), lung disease (yes, no), and bone/joint diseases (yes, no). Household features like religion (Hindu, Non-Hindu); caste/tribe (scheduled tribe, ST; scheduled caste, SC; other backward classes, OBC; non-SC/ST/OBC); monthly per capita consumption expenditure (MPCE) quintile (poorest, poorer, middle, richer, richest); and community-level characteristics such as residence (rural, urban) and geographical region (North, Central, East, North East, West, South) were included in the analysis. Health insurance (yes, no) was used to predict treatment-seeking for insomnia.

Although there are different ways to classify the elderly population, several studies have classified elderly adults as youngest-old (below 75, middle-old (75–84), and those aged over 85 as oldest-old [22, 23]. However, as more than three-fourths of the present study sample was in the youngest old category, i.e., below 75, we clubbed the middle-old and oldest-old together for meaningful analysis. The participants who ever watched television/listened to the radio were considered to have mass media exposure. The perception and understanding of sleep vary by religion in the country; thus, religion was included in the analysis. Caste/tribal status continues to play a major role in healthcare awareness and its utilization- backward castes being worst affected. The STs are communities made up of indigenous people who are marginalized because of their geographic isolation in primarily rural areas. SCs, also called *Dalits*, are historically stigmatized groups who often experience social, educational, and economic exclusion [24, 25]. Other Backward Classes (OBC) is a collective term for castes that are thought to be socially and educationally disadvantaged but do not fit under the STs and SCs categories [24]. Non-ST/SC/OBCs are comprised of people who are not socially or economically disadvantaged [25]. MPCE directly measures household economic well-being without income data [26]. The stratification based on economic condition further holds significance for targeted policy and program measures aiming at the welfare of the disadvantaged population. Overall, the included variables have been proven to influence the health and welfare of the Indian population.

### Statistical analysis

Participants’ socioeconomic, demographic, and health profiles were presented using descriptive analysis. Bivariate analyses examined individual relationships between predictors and the outcome variables. Binary logistic regression was used to assess the adjusted association of the predictor variables with insomnia and treatment-seeking. Outcomes in the regression model included insomnia and treatment-seeking, both classified as ‘1’ = yes and ‘0’ = no. Predictor variables included in the regression analysis were finalized after checking collinearity through the variance inflation factor (VIF) method. The Pearson χ^2^ goodness-of-fit test was used to determine the fit. National-level individual sample weight was used to adjust for non-responses. Stata (v 16.0) was used for analyses, with a 5% significance level.

## Results

### Sample characteristics

**Fig. 1:**
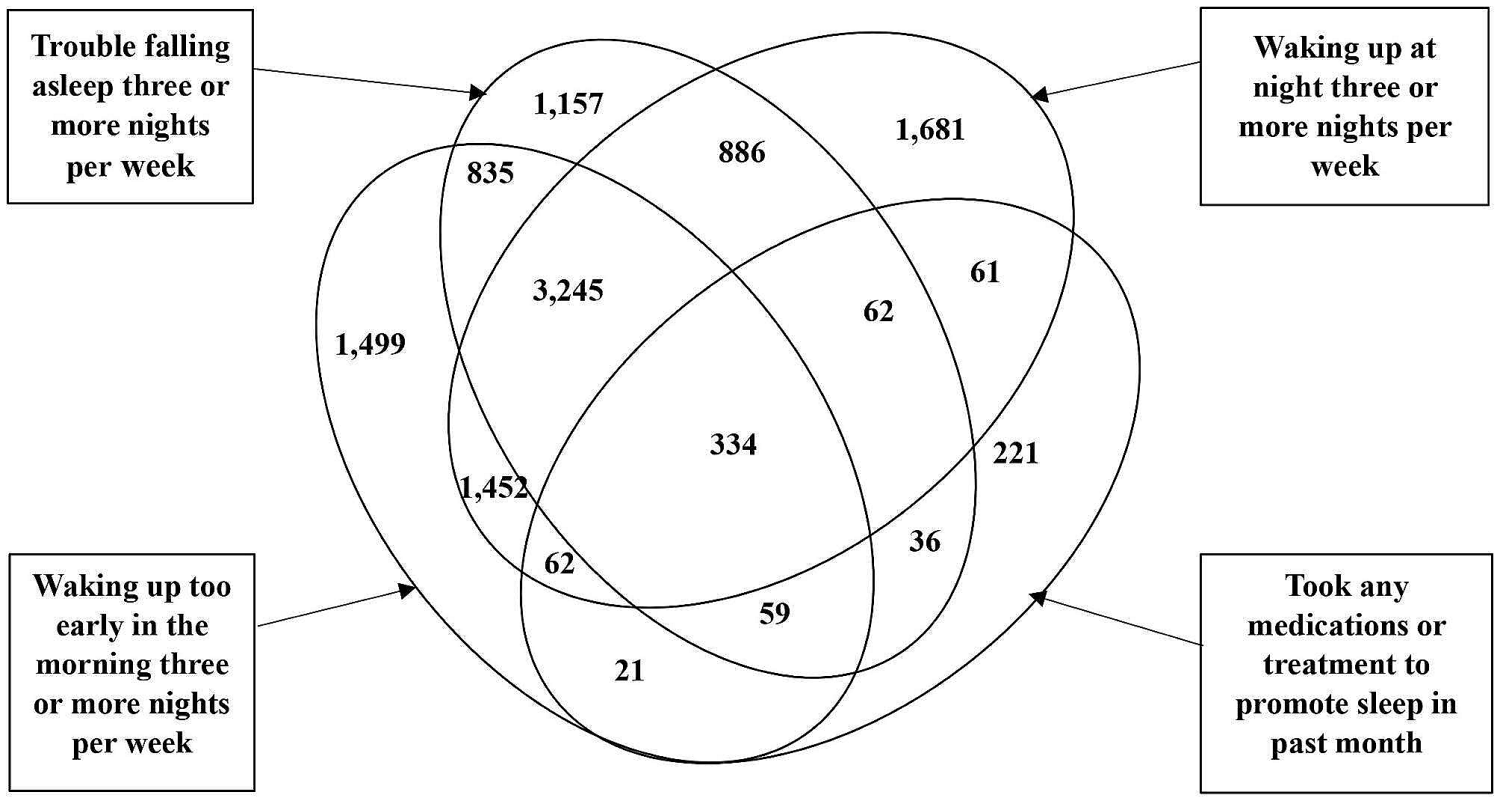
Number of older adults with specific and overlapping insomnia symptoms, India, 2017-18

Table 1 presents the study participants’ socioeconomic, demographic, and health profiles. 77% of participants were aged 60–74, and 23% were aged 75–116. Of the participants, 53% were females, 68% were illiterates, 46% engaged in no/light physical activity, 14% smoked, 35% had CVDs, 8% had lung diseases, and 20% had bone/joint diseases, and 18% were covered by health insurance. Additionally, 82% were Hindu, 45% OBCs, and 71% resided in rural areas. 37% of participants had insomnia. Figure 1 depicts a Venn diagram describing the number of respondents with specific and overlapping insomnia symptoms used in the study.

**Table 1:** Percentage distribution of older adults and those suffering from insomnia based on socioeconomic, demographic, and health related characteristics, India, 2017-18

Characteristics	%Distribution of older adults	% Distribution of older adults suffering from insomnia		χ2 value	Number of older adults
**Age group**				103.85***	
60–74	77.3		35.6		24,308
75–116	22.7		40.7		7156
**Sex**				266.74***	
Male	47.4		32.0		14,931
Female	52.6		41.0		16,533
**Living arrangement**				188.52***	
Living alone/with distant relatives or non-relatives	11.4		40.6		3592
Living with spouse and children/ with distant relatives or non-relatives	61.0		34.6		19,176
Living with children and with distant relatives or non-relatives	27.6		39.8		8696
**Education**				144.24***	
Illiterate	68.0		39.6		21,381
Literate	32.0		30.6		10,083
**Smoking status** ^**a**^				4.09*	
Yes	13.7		37.4		4297
No	85.2		34.5		26,808
**Mass media exposure** ^**a**^				85.64***	
Yes	45.7		33.7		14,393
No	52.5		39.8		16,511
**Physical activity** ^**a**^				189.56***	
No / Light	44.7		41.4		14,060
Moderate	31.1		35.3		9782
Vigorous	23.1		30.6		7262
**BMI** ^**a**^				35.33***	
Underweight	23.5		40.3		7406
Normal weight	45.1		35.7		14,208
Obese	19.5		35.5		6148
**CVDs** ^**a, b**^				308.96***	
Yes	35.2		42.4		11,058
No	64.3		34.0		20,217
**Lung Diseases** ^**a, c**^				171.78***	
Yes	8.4		47.2		2651
No	91.0		36.0		28,627
**Bone/joint diseases** ^**a, d**^				378.09***	
Yes	19.6		48.1		6164
No	79.8		34.2		25,115
**Religion**				70.56***	
Hindu	18.0		37.1		25,871
Non-Hindu	80.7		34.8		5593
**Social-group**				179.59***	
ST	8.1		32.8		2556
SC	18.9		39.2		5949
OBC	45.2		36.7		14,231
Non-SC/ST/OBC	27.8		36.2		8728
**MPCE Quintile**				8.25	
Poorest	21.7		37.0		6829
Poorer	21.7		36.1		6832
Middle	20.9		36.1		6590
Richer	19.2		36.8		6038
Richest	16.5		38.0		5175
**Residence**				33.96***	
Rural	70.6		37.8		22,196
Urban	29.4		34.2		9268
**Region** ^**e**^				211.09***	
North	12.6		38.2		3960
Central	20.9		37.8		6593
East	23.6		37.2		7439
North East	3.0		32.2		935
West	17.2		36.4		5401
South	22.7		35.3		7136
**Total**	**100.00**		**36.7**		**31,464**

**Note**: Significant at * *p* < 0.05, ** *p* < 0.01, *** *p* < 0.001

• BMI = Body mass index; CVD = Cardiovascular disease; ST = Scheduled tribe; SC = Scheduled caste; OBC = Other backward classes; MPCE = Monthly per capita consumption expenditure

• ^a^ Total may not add to “n” due to missing cases

• ^b^ Cardiovascular diseases include hypertension, heart diseases and stroke (any one or more)

•^c^ Lung diseases include chronic obstructive pulmonary disease (COPD), asthma and bronchitis (any one or more)

•^d^ Bone/joint diseases include arthritis, rheumatism and osteoporosis (any one or more)

• ^e^ Region include North (Chandigarh, Delhi, Haryana, Himachal Pradesh, Jammu & Kashmir, Punjab, Rajasthan, Uttarakhand), Central (Chhattisgarh, Madhya Pradesh, Uttar Pradesh), East (Bihar, Jharkhand, Odisha, West Bengal), North East (Arunachal Pradesh, Assam, Manipur, Meghalaya, Mizoram, Nagaland, Tripura), West (Dadra & Nagar Haveli, Daman & Diu, Goa, Gujarat, Maharashtra) and South (Andaman & Nicobar Islands, Andhra Pradesh, Karnataka, Lakshadweep, Puducherry, Tamil Nadu, Telangana)

### Socioeconomic, health, and demographic differential in the prevalence of insomnia among older adults

41% of women and 32% of men had insomnia. 40% of illiterate and 31% of literate participants had insomnia. Of participants, 41% of those engaged in no/low physical activity had insomnia, compared to 31% among those who engaged in vigorous physical activity. 42% of participants with CVDs had insomnia, versus 34% of those without CVDs. 47% of those with lung diseases had insomnia, compared with 36% of those without lung diseases. 48% of those with bone/joint diseases had insomnia, which was only 34% among those who did not have these diseases.

### Determinants of insomnia among older adults

Adjusted odds ratios (ORs) revealed that the likelihood of insomnia increased with age (Table 2). Female participants (OR:1.38, CI: 1.30–1.47), participants living alone/with distant relatives or non-relatives (OR:1.18, CI: 1.08–1.29), and underweight participants (OR:1.16, CI:1.08–1.23) had higher odds of insomnia than their respective counterparts. Literate participants (OR: 0.87, CI: 0.81–0.92), those exposed to mass media (OR:0.88, CI: 0.83–0.94), those engaged in moderate physical activity (OR: 0.80, CI: 0.74–0.84) and vigorous physical activity (OR:0.80, CI;0.75–0.86), non-Hindu (OR: 0.85, CI: 0.80, 0.91) and urban participants (OR:0.86, CI: 0.80–0.91) were less likely to have insomnia than their counterparts. Participants with CVDs (OR:1.49, CI: 1.41–1.57), lung diseases (OR:1.52, CI: 1.39–1.67), and bone/joint diseases (OR:1.60, CI:1.50–1.71) had higher odds of insomnia than their respective counterparts. The SC (OR: 1.33, CI: 1.20–1.47), OBC (OR: 1.38, CI: 1.27–1.514), and non-SC/ST/OBC (OR:1.33, CI:1.21–1.46) participants were more likely to have insomnia than STs. Compared with the participants from the southern region, those from the northern (OR:1.31, CI: 1.21–1.42), central (OR:1.24, CI: 1.13–1.35), and western (OR:1.13, CI: 1.03–1.23) regions were more likely to have insomnia. The results were almost similar for the unadjusted odds ratio.

**Table 2:** Odds Ratio (OR) for insomnia among older adults aged 60 + years, India, 2017-18

Characteristics	Adjusted OR (95% CI)	Unadjusted OR (95% CI)
**Age group**		
60–74®	1.00	1.00
75–116	1.10** (1.03, 1.17)	1.33*** (1.26, 1.41)
**Sex**		
Male®	1.00	1.00
Female	1.38*** (1.30, 1.47)	1.47*** (0.43, 0.46)
**Living arrangement**		
Living with spouse and children/ with distant relatives or non-relatives®	1.00	1.00
Living alone/with distant relatives or non-relatives	1.18***(1.08, 1.29)	1.40*** (1.30, 1.51)
Living with children and with distant relatives or non-relatives	1.14*** (1.07, 1.21)	1.39*** (1.32, 1.47)
**Education**		
Illiterate®	1.00	1.00
Literate	0.87*** (0.81, 0.92)	0.74*** (0.70, 0.78)
**Smoking status** ^**a**^		
No®	1.00	1.00
Yes	1.08 (1.00, 1.17)	0.93* (0.87, 1.00)
**Mass media exposure** ^**a**^		
No®	1.00	1.00
Yes	0.88*** (0.83, 0.94)	0.80*** (0.77, 0.84)
**Physical activity** ^**a**^		
No / light®	1.00	1.00
Moderate	0.80*** (0.74, 0.84)	0.79*** (0.75, 0.83)
Vigorous	0.80*** (0.75, 0.86)	0.67*** (0.63, 0.71)
**BMI** ^**a**^		
Normal weight®	1.00	1.00
Underweight	1.16*** (1.08, 1.23)	1.20*** (1.13, 1.28)
Obese	0.96 (0.90, 1.02)	1.08* (1.02, 1.15)
**CVDs** ^**a, b**^		
No®	1.00	1.00
Yes	1.49*** (1.41, 1.57)	1.53*** (1.46, 1.60)
**Lung Diseases** ^**a, c**^		
No®	1.00	1.00
Yes	1.52*** (1.39, 1.67)	1.75*** (1.60, 1.90)
**Bone/joint diseases** ^**a, d**^		
No®	1.00	1.00
Yes	1.60*** (1.50, 1.71)	1.78*** (1.68, 1.89)
**Religion**		
Hindu®	1.00	1.00
Non-Hindu	0.85*** (0.80, 0.91)	0.80*** (0.76, 0.84)
**Social-group**		
ST®	1.00	1.00
SC	1.33*** (1.20, 1.47)	1.62*** (1.49, 1.76)
OBC	1.38*** (1.27, 1.51)	1.58*** (1.47, 1.69)
Non-SC/ST/OBC	1.33*** (1.21, 1.46)	1.50*** (1.39, 1.62)
**MPCE Quintile**		
Poorest®	1.00	1.00
Poorer	1.01 (0.93, 1.09)	1.02 (0.95, 1.09)
Middle	0.99 (0.92, 1.08)	1.00 (0.93, 1.07)
Richer	1.03 (0.94, 1.11)	1.03 (0.96, 1.11)
Richest	1.07 (0.98, 1.17)	1.10* (1.02, 1.18)
**Residence**		
Rural®	1.00	1.00
Urban	0.86*** (0.80, 0.91)	0.86*** (0.82, 0.91)
**Region** ^**e**^		
South®	1.00	1.00
North	1.31*** (1.21, 1.42)	1.28*** (1.19, 1.37)
Central	1.24*** (1.13, 1.36)	1.19*** (1.10, 1.29)
East	1.05 (0.97, 1.15)	1.04 (0.97, 1.12)
North East	0.90 (0.81, 1.00)	0.68*** (0.63, 0.75)
West	1.13** (1.03, 1.23)	1.05 (0.97, 1.14)

**Note**: ® Reference category, Significant at * *p* < 0.05, ** *p* < 0.01, *** *p* < 0.001

• BMI = Body mass index; CVD = Cardiovascular disease; ST = Scheduled tribe; SC = Scheduled caste; OBC = Other backward classes; MPCE = Monthly per capita consumption expenditure

• ^a^ Total may not add to “n” due to missing cases

• ^b^ Cardiovascular diseases include hypertension, heart diseases and stroke (any one or more)

• ^c^ Lung diseases include chronic obstructive pulmonary disease (COPD), asthma and bronchitis (any one or more)

• ^d^ Bone/joint diseases include arthritis, rheumatism and osteoporosis (any one or more)

• ^e^ Region include North (Chandigarh, Delhi, Haryana, Himachal Pradesh, Jammu & Kashmir, Punjab, Rajasthan, Uttarakhand), Central (Chhattisgarh, Madhya Pradesh, Uttar Pradesh), East (Bihar, Jharkhand, Odisha, West Bengal), North East (Arunachal Pradesh, Assam, Manipur, Meghalaya, Mizoram, Nagaland, Tripura), West (Dadra & Nagar Haveli, Daman & Diu, Goa, Gujarat, Maharashtra) and South (Andaman & Nicobar Islands, Andhra Pradesh, Karnataka, Lakshadweep, Puducherry, Tamil Nadu, Telangana)

### Determinants of treatment-seeking of older adults with insomnia

3% of participants sought treatment for insomnia in the month preceding the survey (figure not shown). Of the participants with insomnia, after adjusting for the effects of the model’s predictors, participants with mass media exposure (OR: 0.85, CI:0.72-1.00) had lesser odds of seeking treatment than those without mass media exposure (Table 3). Participants who engaged in moderate physical activity (OR: 0.76, CI:0.64–0.89) and vigorous physical activity (OR: 0.96, CI:0.81–1.13) were less likely to seek treatment than those with no or light physical activity. Female participants were more likely to seek treatment (OR: 1.29, CI:1.80–1.52) than male participants. Participants with CVDs (OR:2.65, CI:2.27–3.09), lung disease (OR: 1.59, CI:1.28–1.96), and bone/joint disease (OR: 1.36, CI:1.16–1.60) had higher odds of seeking treatment than their counterparts. Participants from non-Hindu religions (OR:1.22, CI:1.04–1.44) were more likely to seek treatment than those from the Hindu religion. Participants belonging to SC (OR: 1.48, CI:1.05–2.08), OBC (OR:1.70, CI:1.25–2.31), and non-ST/SC/OBC (OR:1.78, CI:1.30–2.42) were more likely to seek treatment than those from ST. Participants from the richer (OR: 1.59, CI:1.24–2.03) and richest MPCE quintiles (OR:1.85, CI:1.44–2.37) were more likely to seek treatment than those from the poorest MPCE quintiles. Urban participants had higher odds (OR:1.30, CI: 1.11–1.53) of seeking treatment than rural participants. Participants from the northern (OR:1.71, CI: 1.38–2.12) and eastern (OR: 1.42, CI: 1.12–1.80) regions were more likely to seek treatment than those from the southern region. The results were almost similar for the unadjusted odds ratio.

**Table 3:** Odds ratio (OR) of treatment-seeking for insomnia in the past one month among older adults aged 60 + years, India, 2017-18

Characteristics	Adjusted OR (95% CI)	Unadjusted OR (95% CI)
**Age group**		
60–74®	1.00	1.00
75–116	1.04 (0.87, 1.24)	1.28** (1.10, 1.48)
**Sex**		
Male®	1.00	1.00
Female	1.29** (1.08, 1.52)	1.34*** (1.18, 1.53)
**Living arrangement**		
Living with spouse and children/ with distant relatives or non-relatives®	1.00	1.00
Living alone/with distant relatives or non-relatives	0.77* (0.59, 1.00)	0.85 (0.68, 1.07)
Living with children and with distant relatives or non-relatives	0.88 (0.74, 1.05)	1.12 (0.97, 1.29)
**Education**		
Illiterate®	1.00	1.00
Literate	1.08 (0.91, 1.28)	1.35*** (1.19, 1.54)
**Smoking status** ^**a**^		
No®	1.00	1.00
Yes	0.90 (0.71, 1.15)	0.68*** (0.55, 0.84)
**Mass media exposure** ^**a**^		
No®	1.00	1.00
Yes	0.85* (0.72, 1.00)	1.14* (1.00, 1.30)
**Physical activity** ^**a**^		
No / light®	1.00	1.00
Moderate	0.76*** (0.64, 0.89)	0.73*** (0.63, 0.84)
Vigorous	0.60*** (0.49, 0.75)	0.43*** (0.35, 0.52)
**BMI** ^**a**^		
Normal weight®	1.00	1.00
Underweight	0.89 (0.72, 1.10)	0.68*** (0.56, 0.83)
Obese	0.96 (0.81, 1.13)	1.48*** (1.27, 1.72)
**CVDs** ^**a, b**^		
No®	1.00	1.00
Yes	2.65*** (2.27, 3.09)	3.51*** (3.06, 4.02)
**Lung Diseases** ^**a, c**^		
No®	1.00	1.00
Yes	1.59*** (1.28, 1.96)	1.90*** (1.56, 2.29)
**Bone/joint diseases** ^**a, d**^		
No®	1.00	1.00
Yes	1.36*** (1.16, 1.60)	1.78*** (1.54, 2.06)
**Health insurance** ^**a**^		
No®	1.00	1.00
Yes	0.96 (0.80, 1.15)	0.77** (0.65, 0.91)
**Religion**		
Hindu®	1.00	1.00
Non-Hindu	1.22* (1.04, 1.44)	1.25** (1.09, 1.43)
**Social-group**		
ST®	1.00	1.00
SC	1.48* (1.05, 2.08)	1.80*** (1.36, 2.40)
OBC	1.709*** (1.25, 2.31)	2.05*** (1.60, 2.64)
Non-SC/ST/OBC	1.78*** (1.30, 2.42)	3.17*** (2.47, 4.06)
**MPCE Quintile**		
Poorest®	1.00	1.00
Poorer	1.10 (0.85, 1.43)	1.24 (097, 1.57)
Middle	1.22 (0.95, 1.58)	1.44** (1.14, 1.83)
Richer	1.59*** (1.24, 2.03)	2.09*** (1.68, 2.61)
Richest	1.85*** (1.44, 2.37)	2.65*** (2.14, 3.28)
**Residence**		
Rural®	1.00	1.00
Urban	1.30*** (1.11, 1.53)	1.61*** (1.41, 1.83)
**Region** ^**e**^		
South®	1.00	1.00
North	1.71*** (1.38, 2.12)	1.85*** (1.55, 2.22)
Central	1.18 (0.89, 1.56)	0.77* (0.60, 0.98)
East	1.42** (1.12, 1.80)	1.14 (0.93, 1.39)
North East	0.93 (0.66, 1.29)	0.59*** (0.44, 0.78)
West	1.19 (0.93, 1.52)	1.08 (0.87, 1.35)

**Note**: ® Reference category, Significant at * *p* < 0.05, ** *p* < 0.01, *** *p* < 0.001

• BMI = Body mass index; CVD = Cardiovascular disease; ST = Scheduled tribe; SC = Scheduled caste; OBC = Other backward classes; MPCE = Monthly per capita consumption expenditure

• ^a^ Total may not add to “n” due to missing cases

• ^b^ Cardiovascular diseases include hypertension, heart diseases and stroke (any one or more)

• ^c^ Lung diseases include chronic obstructive pulmonary disease (COPD), asthma and bronchitis (any one or more)

• ^d^ Bone/joint diseases include arthritis, rheumatism and osteoporosis (any one or more)

• ^e^ Region include North (Chandigarh, Delhi, Haryana, Himachal Pradesh, Jammu & Kashmir, Punjab, Rajasthan, Uttarakhand), Central (Chhattisgarh, Madhya Pradesh, Uttar Pradesh), East (Bihar, Jharkhand, Odisha, West Bengal), North East (Arunachal Pradesh, Assam, Manipur, Meghalaya, Mizoram, Nagaland, Tripura), West (Dadra & Nagar Haveli, Daman & Diu, Goa, Gujarat, Maharashtra) and South (Andaman & Nicobar Islands, Andhra Pradesh, Karnataka, Lakshadweep, Puducherry, Tamil Nadu, Telangana)

## Discussion

The study found a sizable number of Indian older adults with insomnia, and the prevalence varied considerably by their socioeconomic, demographic, and health status. There is substantial regional variation in the prevalence of insomnia and treatment-seeking. Increasing age, female gender, not living with a spouse, illiteracy, chronic health conditions, low BMI, lack of moderate or vigorous physical activity, lack of exposure to mass media, Hindu religion, non-tribal status, and rural residence were significantly associated with insomnia. Female gender, lack of exposure to mass media, being physically inactive, having chronic health conditions, non-Hindu religion, non-tribal status, economically better condition, and urban residence were significantly linked with treatment-seeking for insomnia in India.

Insomnia was associated with increasing age, consistent with earlier studies in India [3, 7, 17]. A past study revealed that older adults tend to have sleepiness earlier in the evening and wake up earlier in the morning than desired due to the age-related phase advance in their circadian rhythm, which regulates the timing and structure of sleep [27]. Our findings of higher chances of insomnia among female participants conform with a past study, which noted that in addition to hormonal disturbances, social inequities, and societal expectations might also lead to a higher prevalence of poor sleep quality [3]. We found that older adults living alone/with distant relatives or non-relatives had a higher risk of insomnia. A past study also found that living in a “broken family-family associated with divorce, or where members are in conflict with/ estranged from each other” is independently associated with poor quality of sleep [3, 26]. Literate older adults were less likely to have insomnia, consistent with a prior study [7] that found that higher education level is associated with less insomnia as education is related to the general health of the population [28]. Participants with mass media exposure had a lower risk of insomnia, which could be due to the positive role of television or radio in reducing loneliness [3]. This result contradicts earlier studies, which suggested that watching television could cause both direct and indirect sleep problems, such as encroaching media activity on sleep time [29], bright flickering light affecting sleep-inducing hormones [30], and greater physical inactivity associated with television watching [15].

In our study, participants who did not engage in moderate or vigorous physical activity were more likely to have insomnia, similar to a past study that found that more physical activity was associated with better sleep quality [31]. This result strengthens the need to adopt strategic objectives recommended by the World Health Organization, requiring an active society with active people and active environment and systems for reducing the prevalence of physical inactivity [32], which will further reduce the prevalence of non-communicable diseases including insomnia. We found a higher prevalence of insomnia among the underweight participants, which contradicts some earlier studies [33, 34] noting that sleep duration was positively associated with obesity risk. However, another study found associations between low-calorie intake and (a) low sleep-inducing gut peptides like cholecystokinin and (b) higher wake-promoting substances like orexin [35]. Moreover, dietary nutrition has a significant impact on sleep [36]. This result suggests the need for policies and programs providing nutritious food, especially to economically disadvantaged older adults, through the existing programs aimed at the welfare of older persons. For example, aids and assistive living devices are provided to economically-weaker senior citizens through Rashtriya Vayoshri Yojana [37] and these beneficiaries may also be considered for a cash-incentives to address their nutritional requirements.

Chronic diseases were significantly associated with insomnia, consistent with past studies in which participants with CVDs [38], lung diseases [38, 39], and bone/joint diseases [39, 40] were more likely to have insomnia. This can be explained by various pathophysiological causes, such as waking up suddenly with discomfort during sleep, sleep-disrupted breathing, or the onset of nocturia, which can cause repeated awakening and difficulties returning to sleep [41]. We found that insomnia was considerably higher among Hindus, consistent with a previous study [42]. The perception and understanding of sleep vary by religion [43], though more religious adults exhibit healthier sleep outcomes [16]. Hindus divide consciousness into three states: waking, dreaming, and deep sleep, and believe that both dreaming and deep sleep are more critical than waking [43], in contrast to Western culture, which considers waking the most crucial state [44]. Hindus believe only deep sleep, not waking or dreaming, is sleep [45]; this definitional variation may be the reason for the higher prevalence of reported insomnia among Hindus. Insomnia was more common among the non-tribal population, possibly related to differences in work-related activities performed by people from different social groups: work requirements among tribal people often require moderate/ vigorous physical activity [46]. Moreover, higher consumption of alcohol among the tribes [47] may be another possible reason for lower insomnia among the tribal population. In one study, older adults who consumed alcohol were less likely to experience insomnia symptoms than those who did not consume alcohol [48]. Alcohol at bedtime accelerates sleep onset and increases the amount of slow-wave sleep [13]. Alcohol is frequently used as a sleeping aid [49], although effects wane with prolonged use [13]. Insomnia was more common in rural areas, consistent with a past study that noted that high demands for physical activity frequently led to musculoskeletal pain or sickness and decreased sleep quality among rural workers [50]. Insomnia was significantly lower among participants from the southern region, perhaps due to better health infrastructure and literacy [51].

Treatment-seeking was very limited, which may be related to sleep literacy and treatment access. Literature reveals that patients with insomnia often do not perceive it as requiring medication and thus do not seek treatment [7]. Our findings are consistent with several studies that found women seek treatment more frequently than men, indicating that they are more likely to visit their doctors in general [52, 53]. We found that participants with mass media exposure were less likely to seek treatment; perhaps the amount and type of information offered in the media shape their ideas, attitudes, and perceived norms about insomnia [54]. Participants were less likely to seek treatment if they lived alone/with distant relatives or non-relatives. These participants may have limited financial resources and access to treatment. Consistent with a prior study [55], physically active older adults were less likely to seek treatment as physical exercise is vital in preventing unhealthy outcomes, and medication usage is frequently a result of those health outcomes. Older adults with chronic diseases were more likely to seek treatment, but this may have been because they were more prone to insomnia. We found lower chances of seeking treatment among the tribes. Several past studies [46, 56] found traditional healing is often the first contact of treatment options among the tribes due to a lack of healthcare facilities, non-availability of Western medicine providers, and easy availability of medicinal plants in their local environment. People from households with high MPCE quintiles were more likely to seek treatment, possibly because of better economic conditions and access to health providers. Belief in traditional herbal remedies, which differ from Western medicines, is more common among Hindus [57], leading to low formal treatment seeking. Treatment-seeking was higher among urban residents, possibly due to ease of access to providers in cities and a better understanding of sleep quality and disorders. Treatment-seeking was less prevalent among participants from South India, possibly as they have a lower chance of perceiving themselves as having insomnia.

Medicine is beneficial for treating acute insomnia in the short term; however, its long-term use is controversial [58], although the use of off-label medication to treat insomnia is common [59]. Cognitive behavioral therapy for insomnia (CBT-I) is the first-line treatment for insomnia with well-established efficacy [60]. It consists of a multimodal mix of sleep restriction, cognitive restructuring, relaxation training, sensory control, and sleep education therapies that generally last five weeks [61]. Nevertheless, barriers to the broader use of CBT-I include a lack of qualified therapists and time and money constraints [61]. As a result, hypnotic drugs are still often recommended by doctors and considered necessary in many situations [62]. Problem-solving skills may be used to overcome these problems, and a recent study found that they are also useful for treating insomnia in India. Thus, we might need to integrate problem-solving into CBT-I while working with the Indian population [63].

Regional variation in prevalence and treatment seeking for insomnia suggests customized state-specific programs or policy initiatives to enhance sleep literacy and address the identified modifiable risk factors. However, although the proportion of older adults is increasing in several Asian countries, these countries differ in several population and health indicators, including the literacy rate, mass media exposure, alcohol consumption, religious diversity, and policies and programs for older adults [64]; which might affect insomnia prevalence and treatment-seeking differently.

The strength of this study is the large sample size from a recent nationally representative survey with a robust sampling design. The study provides the prevalence and predictors of insomnia among older adults and treatment-seeking at the national level. The results are contemporary and relevant for targeted programs and policy intervention. However, the cross-sectional nature of the data limits any causal inference about relations between the predictors and insomnia. Data were collected via self-report questionnaires; therefore, the possibility of recall bias and under-reporting cannot be ignored. Moreover, the estimation of insomnia may vary depending on various clinical definitions of insomnia. This data set does not contain sufficient information to assess chronic insomnia, which poses major negative implications. Finally, there was no information in this study about other potential determinants of treatment-seeking, such as sleep literacy and use of nonwestern remedies, and thus these could not be examined.

## Conclusions

A sizable number of older adults have insomnia, and the prevalence varies by their socioeconomic, demographic, and health status. Many modifiable risk factors like low education, chronic health conditions, smoking, being underweight, physical inactivity, and lack of exposure to mass media are identified. Thus, targeted interventions on these modifiable risk factors can be carried out to reduce the burden of insomnia among older adults. Treatment-seeking for Insomnia is inadequate, enhancing the older adult’s vulnerability to various morbidities. Policy and program intervention to raise awareness about insomnia, including early identification and pharmacological and non-pharmacological treatment, will ensure better health and welfare of older adults.

## Data Availability

All data generated or analyzed during this study are included in this published article.

## Abbreviations

OPD: Outpatient Department
LASI: Longitudinal Ageing Study in India
UT: Union Territories
PSU: Primary Sampling Unit
SSU: Secondary Sampling Unit
CAPI: Computer-Assisted Personal Interview
CEB: Census Enumeration Block
ICMR: Indian Council of Medical Research
CVD: Cardiovascular Disease
OBC: Other Backward Classes
SC: Scheduled Caste
ST: Scheduled Tribe
MPCE: Monthly Per Capita Expenditure
VIF: Variance Inflation Factor
OR: Odds Ratio
CI: Confidence Interval
BMI: Body Mass Index
CBT-I: Cognitive Behavioral Therapy for Insomnia
